# Association Between Polymorphisms in Genes Related to Common Adult Diseases and Fetal Growth

**DOI:** 10.4137/cmped.s2154

**Published:** 2009-02-17

**Authors:** Hisao Osada

**Affiliations:** Department of Maternal-fetal Medicine, Chiba University Hospital.

**Keywords:** genetic polymorphism, common adult disease, fetal growth, thrifty genotype, insulin, insulin-like growth factor-II, G protein

## Abstract

A close relationship between size at birth and occurrence of common adult diseases has been reported. As an explanation of this relationship, it has been hypothesized that the thrifty genotypes cause changes in growth efficiency during fetal period and diseases in later life. In the present study, we examined the association of fetal growth with genetic polymorphisms within the *IGF2-INS-TH* region and in the G protein gene. Analysis of the genes in the *IGF2-INS-TH* region suggests that thrifty genotype has the effect of accelerating fetal growth, but at the same time a genomic imprinting mechanism is also involved. Analysis of the G protein β3 subunit gene unveiled that the 825T allele in the mother may exert influence on fetal metabolic environment. By extending the analysis to other genomic regions related to common adult diseases using the same technique, the detailed role of genetic polymorphisms may be elucidated.

## Introduction

It has been suggested that polymorphisms associated with susceptibility to common chronic adult diseases are present in the human genome. Genetic polymorphisms refer to the genetic differences among individuals at the level of DNA sequence. In principle, they do not contribute directly to disease, but they may affect the magnitude of gene expression. Neel^1^ hypothesized that our ancestors acquired the “thrifty genotype”, the genotype that allowed them to survive under condition of low energy consumption during long periods of famine in the history of mankind. However, in times of sustained abundance, the presence of this thrifty genotype confers a disadvantage, predisposing the carrier to common adult diseases.

Etiological studies have proved that birth weight is associated with not only postnatal prognosis but also common adult disorders including type 2 diabetes, dyslipidemia, hypertension, and atherosclerosis.^2^–^4^ In the past decade, research has principally focused on the role of the intrauterine environment. It has been hypothesized that undernutrition *in utero* results in permanent reprogramming of fetal metabolism. In other words, genetic factors were not given a place in the ‘thrifty phenotype’ hypothesis.^5^ Nowadays, however, a significant body of evidence suggests that genetic factors are important common determinants of birth weight and adult diseases such as type 2 diabetes.^6^,^7^ Therefore, it has been proposed that the phenotypes of adult diseases are a reflection of the genotypes and the intrauterine and postnatal environment, with different factors having varying predominance in different individuals.^8^,^9^

Gene knockout animal models have shown the importance of genes encoding insulin (INS), insulin-like growth factors (IGFs) and their receptors, in regulating fetal growth and size at birth.^10^–^12^ In humans, rare mutations in these genes lead to severe intrauterine growth restriction.^13^–^15^ Several common gene variants have also been reported to be associated with alterations in birth size. Casteels et al.^16^ reported an association between birth size and a mitochondrial DNA variant, but only in neonates who changed rank in postnatal growth. Cambien et al.^17^ reported that angiotensin I-converting enzyme gene polymorphism modulates *in utero* growth. With the recent advances in genetic technologies such as single nucleotide polymorphisms (SNPs) analysis, validation of common gene variants on a genome-wide scale has become feasible.

**Figure 1:**
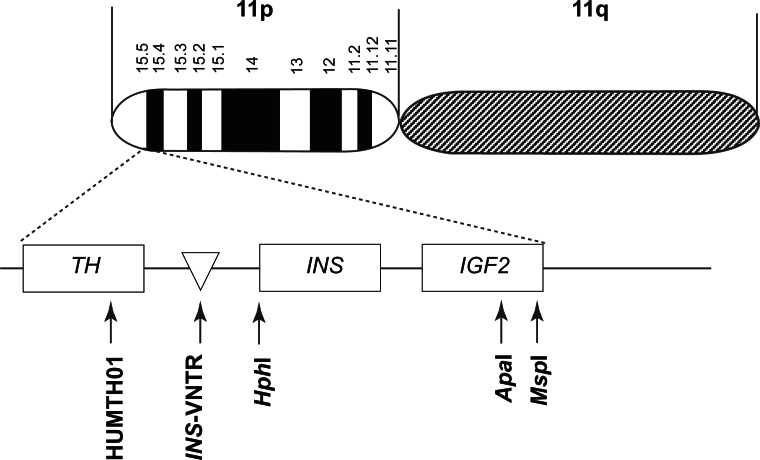
The *IGF2-INS-TH* region on chromosome 11.

Some genes may affect not only fetal but also postnatal growth. Such candidate genes include those encoding INS, the IGFs, their receptors, and the respective post-receptor signaling cascades. The *IGF2-INS-TH* region on human chromosome 11p15.5 has been associated with various common disorders including the metabolic syndrome, type 2 diabetes and coronary heart disease^18^,^19^ (Fig. 1). The genes in this region encode INS, tyrosine hydroxylase (TH) and insulin-like growth factor-II (IGF-II) which is a major fetal growth factor also expressed in adult life. Each gene possesses a number of potential pathways that may impact various metabolic and cardiovascular risk traits. Therefore, we selected this region as the first to analyze in our attempt to validate the thrifty genotype hypothesis.

Numerous hormones, neurotransmitters, chemokines, local mediators, and sensory stimuli exhibit their characteristic effects on cells through binding with G protein-coupled receptors.^20^ G proteins bind to these receptors and play the important role of signal transduction for intracellular reactions that are the basis of physiologic responses in tissues and organisms. While mutations of the G protein gene cause rare diseases such as pseudohypoparathyroidism type I and McCune-Albright syndrome,^21^ a body of evidence has accumulated which suggests an association between balanced polymorphisms of the G protein gene and common chronic diseases.^22^–^26^ We thus expected that this gene would also provide useful information in our validation study.

In the present study, as a model case for genetic linkage between phenotypes during fetal stage and diseases in adulthood, we examined the association of fetal growth with genetic polymorphisms within the *IGF2-INS-TH* region and in the G protein gene.

## Patients and Methods

The subject population consisted of 884 mothers who delivered their neonates after 36 weeks’ gestation. The mothers were non-smoking Japanese women with no remarkable past history and an uneventful pregnant course. Each participant in the study gave informed consent according to a protocol approved by the local institutional review board.

The parameters of fetal growth were birth weight, head circumference, and length. The actual measurements of birth weight, height and head circumference were converted into standard deviation scores (SDS) using the mean and standard deviation values of the standard somatoscopic curves at birth, by sex and by parity (Japanese Ministry of Health and Welfare Research Group 1983, revised in 1994). For instance, birth weight SDS = (birth weight—mean birth weight of neonates for the corresponding gestational week, sex and parity)/(1 SD of neonates for the corresponding gestational week, sex and parity).

Maternal and neonatal genomic DNA was prepared from the peripheral blood of mother and umbilical cord blood of neonate, respectively. Genotyping was performed by polymerase chain reaction (PCR) and PCR-restriction fragment length polymorphism analysis.

## Results

### Analysis of association between *IGF2* polymorphisms and size at birth^27^

Among the SNPs in *IGF2* gene, the +3123*Apa*I and +3580*Msp*I loci were studied. The +3123*Apa*I polymorphism was genotyped for the presence (allele G) or absence (allele A) of the restriction site. GG genotype has been reported to be associated with adult obesity.^28^,^29^ The associations of maternal and neonatal +3123*Apa*I genotypes with SDSs of birth weight are shown in Table 1. There was a significant difference (p = 0.04) in birth weight SDSs among the three neonatal +3123*Apa*I genotypes; AA, AG and GG. GG homozygotes had a mean birth weight SDS of 0.18 higher than that of AA homozygotes (p = 0.01), and heterozygotes showed an intermediate mean value. No significant difference in birth weight SDSs was observed among the three maternal +3123*Apa*I genotypes. There was no significant difference in birth weight SDSs among the +3580*Msp*I genotypes; AA, AG and GG, in both neonatal and maternal samples (Table 1).

### Analysis of association between polymorphisms within the *INS* region and size at birth^30^

We analyzed the genotypes of two polymorphic loci within the *INS* region; −23*Hph*I, a SNP in *INS* gene and HUMTH01, a [AATG]n tetranucleotide repeat microsatellite in *TH* gene.

The −23*Hph*I polymorphisms were genotyped for the presence (A allele) or absence (T allele) of the restriction site. Among the neonates in the present study, the frequencies of the three genotypes for the −23*Hph*I polymorphism were AA: 92.7%, AT: 7.3%, TT: 0%. The associations of neonatal and maternal −23*Hph*I genotypes with SDSs of weight, head circumference, and length at birth are shown in Table 2. There were no significant differences in birth weight SDSs between −23*Hph*I AT and AA genotypes in both neonates and mothers. No significant differences were also observed for head circumference and length SDSs at birth. Based on the segregation patterns of −23*Hph*I alleles from mothers to neonates, neonatal −23*Hph*IAT genotype could be subdivided into 2 types; paternal origin (A/pat T) and maternal origin (A/mat T) of T allele. The birth weight SDSs were significantly greater in −23*Hph*I A/pat T neonates than in −23*Hph*I AA neonates. Similarly, SDSs of head circumference and length at birth were significantly greater in −23*Hph*I A/pat T neonates than in −23*Hph*I AA neonates. In contrast, there were no significant differences in SDSs of the three parameters between −23*Hph*I A/mat T and A/A genotypes.

The HUMTH01 is denoted by the number of tetranucleotide repeats; for example, allele 6 has 6 repeats of AATG. Five (alleles 6, 7, 8, 9, and 10) of the HUMTH01 alleles described previously^31^ were found among the subjects in the present study. In subsequent analyses, therefore, each somatoscopic variable was compared between HUMTH01 10 (+) and 10 (−) genotypes. The associations of neonatal or maternal HUMTH01 genotypes with SDSs of weight, head circumference, and length at birth are shown in Table 3. A significant difference in birth weight SDSs was observed between neonatal HUMTH01 10 (+) and 10 (−) genotypes (p < 0.05). The head circumference and length SDSs at birth were also significantly greater in neonatal HUMTH01 10 (+) genotype than in HUMTH01 10 (−) genotype (p < 0.05). However, there were no significant differences in these SDSs between maternal HUMTH01 10 (+) and 10 (−) genotypes.

The *INS* variable number of tandem repeats (VNTR) locus is located 596 bp 5’ to the *INS* initiation site, and is composed of tandem repetition of a 14–15 bp oligonucleotide consensus sequence.^32^ The *INS*-VNTR has been classified into three distinct classes (I, II and III) according to the number of repeats of the oligonucleotide.^33^ The present study revealed that neonatal −23*Hph*I T allele and HUMTH01 allele10, which are linked to the *INS*–VNTR class III allele, were associated with increased weight, head circumstance, and length at birth. These associations confirm that variation within the *INS* region, most probably at the *INS-*VNTR, influences fetal growth. Furthermore, the finding that the paternally transmitted −23*Hph*I T allele exclusively correlates with increased size at birth indicate the involvement of an imprinting mechanism.

### Analysis of association between G protein β3 subunit C825T polymorphism and size at birth^34^

G protein is composed of α, β, and γ subunits. Siffert et al.^22^ discovered a common polymorphism (C825T) in exon 10 of the gene that encodes the β3 subunit. The splice variant corresponding to the 825T allele synthesizes a protein short of 41 amino acids and one domain. In *in vitro* studies, this truncated protein has been shown to be associated with enhanced activity of G proteins. Furthermore, G protein β3 subunit C825T polymorphism has attracted attention because of its association with common disorders in adults such as hypertension^22^–^25^ and obesity.^26^ From these findings, we expected that G protein β3 subunit C825T polymorphism would also provide useful information in our validation study.

The association between maternal genotypes and neonatal somatoscopic characteristics at birth is shown in Table 4. The SDS values of neonatal head circumference was −0.274 ± 0.783 (mean ± SD) with maternal TT/CT genotypes and −0.008 ± 0.851 with CC genotype, and was significantly smaller in maternal TT/CT genotypes (*p* = 0.028). The mean SDS values of neonatal head circumference were −0.227 and −0.150 for neonatal TT/CT and CC genotypes, respectively, and they were not significantly different.

Therefore, we observed an association of the maternal (but not neonatal) G protein β3 subunit 825T allele with reduced head circumference of the neonate. Although detailed mechanism of this association requires further research, our result suggests that expression of the β3 subunit 825T allele in the mother may exert an influence on fetal metabolic environment.

## Discussion

Using INS resistance as a model, Hattersley et al.^8^ advocated shared genotypes as an explanation for the link between fetal growth and common adult diseases. However, previous studies on the association of the thrifty genotype depicted a rather simplified story that growth regulation during fetal period affects the occurrence of common diseases in later life. For example, Vaessen et al.^35^ analyzed polymorphisms in the promoter region of *IGF1* gene, and reported that alleles other than the wild type are related to lower birth weight and also associated with diabetes mellitus or cardiovascular disease in later life. However, the present study demonstrated that the association of the thrifty genotype is more complicated. Analysis of the genes in the *IGF2-INS-TH* region suggests that thrifty genotype has the effect of accelerating fetal growth, but at the same time a genomic imprinting mechanism is also involved.

IGF-II is a major fetal growth factor also expressed in adult life. The *IGF2* gene is involved in a number of potential pathways that may impact various metabolic and cardiovascular risk traits. A genome scan provided replicated evidence of linkage for abdominal subcutaneous and visceral fat on 11p15.5, especially in the *IGF2* region.^36^ In addition, several studies have demonstrated the presence of differential mRNA expression or transcription of the *IGF2* gene depending on genotypes.^37^,^38^ In the present study, the +3123*Apa*I GG genotype (reported to be related to adult obesity)^28^,^29^ was associated with higher birth weight than the AA genotype.

The *INS*-VNTR class I/III genotype has been shown to be associated with type 1 and 2 diabetes,^39^–^41^ obesity,^42^ and polycystic ovary syndrome.^43^ Other studies showed that the genotype is related to growth in early life.^44^,^45^ Our results indicate that *INS*-VNTR class III correlates with increased size at birth. The *INS*-VNTR class III allele is related to regulated translation of the *INS* gene in the fetal pancreas, and it is speculated that lowered INS secretion causes INS resistance in fetal peripheral tissues. As far as nutrition supply is sufficient, peripheral INS resistance is thought to amplify anabolism of INS and stimulate growth, as is observed in the adolescent period.^41^ Taken together, we conclude that size at birth is influenced by expression of the *INS* gene, or of neighboring genes regulated by the *INS*-VNTR.

Furthermore, the results of the present study is consistent with previous reports of the involvement of genomic imprinting in the association between class III allele and type 2 diabetes and also the fact that *IGF2* gene is a representative maternal imprinting gene. Analysis of *INS*-VNTR in type 2 diabetic parents-offspring trios has demonstrated that susceptibility to the disease was exclusively mediated by paternally derived class III allele.^46^ Only a few studies have assessed the potential role of the parent-of-origin of the *INS*-VNTR allele for fetal growth, and their results remain controversial.^45^,^47^ In the present study, segregation analyses revealed that paternally transmitted *INS*-VNTR class III allele was associated with increased weight, head circumference, and height at birth. Thus our results indicate that *INS*-VNTR class III allele may also affect fetal growth in a parent-specific manner.

On the other hand, analysis of the G protein β3 subunit gene unveiled an entirely different pathway of involvement; that expression of the β3 subunit 825T allele in the mother may exert influence on fetal metabolic environment, perhaps through changes in the maternal intrauterine environment or maternal metabolism. While high glucose level in maternal blood caused by gestational diabetes can lead to fetal macrosomia, a more subtle change in blood glucose may also affect fetal weight gain, as demonstrated in a study of mothers with rare genetic defects of the glucokinase gene.^48^ It has been reported that the mitochondrial 16,189 variant, which is maternally transmitted, is associated with size at birth as well as type 2 diabetes.^49^ A common variation in exclusively maternally expressed *H19* gene also has been associated with birth size in smaller first-born infants.^50^ These data suggest that not only fetal genes but also genes that regulate the maternal uterine environment could be important in determining size at birth.

Recently, the concept of “developmental origins of health and disease (DOHaD),”^51^–^53^ which hypothesizes that health and diseases in adulthood are determined by the nutrition status in fetal and infantile stages, has attracted much attention. How the association of the thrifty genotype with fetal growth may contribute to the DOHaD hypothesis should be examined. The risk of diabetes with obesity is particularly evident in ethnic populations with a dramatic improvement in nutrition, such as immigrants.^54^ In addition to metabolic changes programmed by fetal and early postnatal poor nutrition, this tendency may reflect a high prevalence of thrifty genotype, which has resulted in improved survival but the same metabolic adaptation may increase the risk of adult diseases. Evaluation of the genetic factors involved in the association between size at birth and adult disease may provide us with tools to identify the highest risk groups and to introduce early targeted interventions.

The study on the relationship between genetic polymorphisms and fetal growth has only started. In the future, by extending the analysis to other genomic regions related to common adult diseases using the same technique, the detailed role of genetic polymorphisms may be elucidated. Finally, as a future outlook, the utilization of genetic polymorphism data to customize nutrition management programs for individual mothers and fetuses may be realized.

## Figures and Tables

**Table 1:** Association between Genotypes of IGF2 SNPs and birth weight SDSs.

**SNPs**	**Genotype**		**n**	**Weight^a^**		**ANOVA**
				**Mean**	**SD**	***p***
+3123*Apa*I	Neonate	AA	157	−0.195	0.805	
		AG	411	−0.070	0.848	0.04
		GG	316	−0.011	0.887	
	Mother	AA	151	−0.125	0.800	
		AG	417	−0.070	0.847	0.47
		GG	316	−0.023	0.896	
+3580*Msp*I	Neonate	AA	44	−0.178	0.842	
		AG	255	0.032	0.864	0.09
		GG	585	−0.096	0.853	
	Mother	AA	34	0.060	0.772	
		AG	254	−0.086	0.833	0.64
		GG	596	−0.060	0.872	

a Represented by standard deviation scores (SDS). Neonatal +3123*Apa*l AA vs. GG; P = 0.01 for birth weight SDS.

p < 0.05 are underlined.

**Table 2:** Association between −23*Hph*I genotypes and size at birth.

**Genotype**		**n**	**Weight^a^**		***p***	**Head circumference^a^**		***p***	**Height^a^**		***p***
			**Mean**	**SD**		**Mean**	**SD**		**Mean**	**SD**	
Neonate	AT	38	0.112	0.778	0.073^d^	−0.025	0.864	0.077^d^	−0.041	0.708	0.277^d^
	A/pat T^b^	17	0.252	0.794	0.045^d^	0.217	0.703	0.006^d^	0.252	0.665	0.016^d^
	A/mat T^c^	21	−0.001	0.764	0.509^d^	−0.222	0.946	0.981^d^	−0.278	0.665	0.507^d^
	AA	482	−0.109	0.844		−0.270	0.739		−0.193	0.829	
Mother	AT	38	0.041	0.823	0.225^e^	−0.179	0.743	0.621^e^	−0.253	0.727	0.608^e^
	AA	482	−0.103	0.842		−0.258	0.752		−0.177	0.829	

e Represented by standard deviation scores (SDS).

b Paternal origin of T allele.

c Maternal origin of T allele.

d Versus neonatal A/A genotype.

e Versus maternal A/A genotype.

There was no case with homozygous status of −*22Hph*I T allele.

p < 0.05 are underlined.

**Table 3:** Association between HUMTH01 genotypes and size at birth.

**Genotype**		**n**	**Weight^a^**		***p***	**Head circumference^a^**		***p***	**Height^a^**		***p***
			**Mean**	**SD**		**Mean**	**SD**		**Mean**	**SD**	
Neonate	10 (+)	44	0.138	0.742	0.031^b^	−0.002	0.870	0.048^b^	0.033	0.634	0.037^b^
	10 (−)	476	−0.014	0.847		−0.275	0.735		−0202	0.834	
Mother	10 (+)	45	0.067	0.810	0.100^c^	−0.144	0.786	0.560^c^	−0.116	0.685	0.401^c^
	10 (−)	475	−0.108	0.843		−0.262	0.747		−0.188	0.833	

a Represented by standard deviation scores (SDS).

b Versus neonatal 10 (−) genotype.

c Versus maternal 10 (−) genotype.

p < 0.05 are underlined.

**Table 4:** Association between G protein β3 subunit C825T polymorphism and size at birth.

**Genotype**		**n**	**Weight^a^**		***p***	**Head circumference^a^**		***p***	**Height^a^**		***p***
			**Mean**	**SD**		**Mean**	**SD**		**Mean**	**SD**	
Mother	TT/CT	258	0.002	0.856	0.591^b^	−0.274	0.783	0.028^b^	−0.201	0.076	0.832^b^
	CC	84	0.071	0.864			−0.008	0.851	−0.232	0.128	
Neonate	TT/CT	241	0.037	0.854	0.648^c^	−0.227	0.754	0.510^c^	−0.151	0.077	0.164^c^
	CC	101	−0.020	0.868		−0.150	0.929		−0.351	0.121	

a Represented by standard deviation scores (SDS).

b Versus maternal CC genotype.

c Versus neonatal CC genotype. p < 0.05 are underlined.

## Abbreviations

SNPs: single nucleotide polymorphisms;
INS: insulin;
IGFs: insulin-like growth factors;
TH: tyrosine hydroxylase;
IGF-II: insulin-like growth factor-II;
SDS: standard deviation scores;
PCR: polymerase chain reaction;
VNTR: variable number of tandem repeats;
DOHaD: developmental origins of health and disease.
